# Factors that influence uptake of routine postnatal care: Findings on women’s perspectives from a qualitative evidence synthesis

**DOI:** 10.1371/journal.pone.0270264

**Published:** 2022-08-12

**Authors:** Emma Sacks, Kenneth Finlayson, Vanessa Brizuela, Nicola Crossland, Daniela Ziegler, Caroline Sauvé, Étienne V. Langlois, Dena Javadi, Soo Downe, Mercedes Bonet

**Affiliations:** 1 Department of International Health, Johns Hopkins School of Public Health, Baltimore, Maryland, United States of America; 2 School of Community Health and Midwifery, University of Central Lancashire, Preston, United Kingdom; 3 Department of Reproductive Health and Research, World Health Organization, Genève, Switzerland; 4 Centre Hospitalier de l’Universite de Montreal, Montreal, Canada; 5 Partnership for Maternal, Newborn, and Child Health, World Health Organization, Genève, Switzerland; 6 Department of Social and Behavioral Sciences, Harvard T.H. Chan School of Public Health, Boston, Massachusetts, United States of America; Jhpiego, UNITED STATES

## Abstract

**Background:**

Effective postnatal care is important for optimal care of women and newborns–to promote health and wellbeing, identify and treat clinical and psychosocial concerns, and to provide support for families. Yet uptake of formal postnatal care services is low and inequitable in many countries. As part of a larger study examining the views of women, partners, and families requiring both routine and specialised care, we analysed a subset of data on the views and experiences of women related to routine postnatal care.

**Methods:**

We undertook a qualitative evidence synthesis, using a framework analysis approach. We included studies published up to December 2019 with extractable qualitative data, with no language restriction. We focused on women in the general population and their accounts of routine postnatal care utilization. We searched MEDLINE, PUBMED, CINAHL, EMBASE, EBM-Reviews, and grey literature. Two reviewers screened each study independently; inclusion was agreed by consensus. Data abstraction and scientific quality assessment were carried out using a study-specific extraction form and established quality assessment tools. The analysis framework was developed *a priori* based on previous knowledge and research on the topic and adapted. Due to the number of included texts, the final synthesis was developed inductively from the initial framework by iterative sampling of the included studies, until data saturation was achieved. Findings are presented by high versus low/middle income country, and by confidence in the finding, applying the GRADE-CERQual approach.

**Findings:**

Of 12,678 papers, 512 met the inclusion criteria; 59 articles were sampled for analysis. Five themes were identified: access and availability; physical and human resources; external influences; social norms; and experience of care. High confidence study findings included the perceived low value of postnatal care for healthy women and infants; concerns around access and quality of care; and women’s desire for more emotional and psychosocial support during the postnatal period. These findings highlight multiple missed opportunities for postnatal care promotion and ensuring continuity of care.

**Conclusions:**

Factors that influence women’s utilization of postnatal care are interlinked, and include access, quality, and social norms. Many women recognised the specific challenges of the postnatal period and emphasised the need for emotional and psychosocial support in this time, in addition to clinical care. While this is likely a universal need, studies on mental health needs have predominantly been conducted in high-income settings. Postnatal care programmes and related research should consider these multiple drivers and multi-faceted needs, and the holistic postpartum needs of women and their families should be studied in a wider range of settings.

**Registration:**

This protocol is registered in the PROSPERO database for systematic reviews: CRD42019139183.

## Background

Postnatal care (PNC) is a fundamental component of the maternal, newborn and child care continuum, and contributes to reducing maternal and neonatal morbidity and mortality and improving overall health and wellbeing [1–3]. It is generally defined as the care provided during the postnatal period, beginning immediately after childbirth and up to six weeks (42 days) after birth [1] or beyond [4]. PNC represents a set of healthcare services designed to promote the health of women and newborns; it includes risk identification, preventive measures, health education and promotion, and management or referral for complications. Postnatal care not only improves mortality and clinical care, but also affects the satisfaction and experience of health care users; understanding the experiences and needs of women and their families with regard to postnatal care can improve utilization and positive experiences. The World Health Organisation (WHO) recommends that all women and newborns receive postnatal care in the first 24 hours following childbirth, regardless of where the birth occurs, and subsequent postnatal check-ups in the first six weeks [5].

Nevertheless, postnatal care ranks among the lowest coverage of maternal and child health services interventions; after facility discharge, only 31% of women and 13% of newborns receive a postnatal check [6, 7]. Previous studies have also identified important socioeconomic and geographic inequities in access to and utilisation of postnatal care services [8].

Over the last two decades, there have been multiple contributions to a large and growing canon of literature on facilitators and barriers to maternity care, including recent systematic reviews [9–11]. However, most of these studies have focused on care-seeking for intrapartum care and immediate PNC (within 24 hours), and not later (e.g. post discharge) postnatal care [12–14]. Much of the literature on maternity care focuses on facilitators and barriers to utilization [15–18] but, as low quality care has recently been associated with a potentially higher attributable risk of mortality than lack of access [19], studies have begun to examine perceived and actual quality of care, including disrespect and abuse at facilities, as contributing factors to low utilisation of maternal health services [15–18]. Very few studies have examined the impact of mistreatment or disrespect of newborns as discouraging factors for uptake of postnatal care, but recent studies have demonstrated the importance of satisfaction with maternal and neonatal care on subsequent care utilization [20, 21].

This paper presents the results of a sub-set of the data from a qualitative evidence synthesis designed to explore the views and experiences of women, their partners, families and communities in the postnatal period, and factors that influence uptake of routine postnatal care. For this analysis, our aim was to assess the views and experiences of women in the general population in accessing routine postnatal care for themselves and their infants.

## Methods

We included qualitative or mixed-methods studies where the focus was the views of women in the general population (i.e. excluding sub-populations such as adolescents or migrants) on factors that influence uptake of routine postnatal care (i.e. those without additional postnatal needs due to comorbidities or identified medical risk), irrespective of parity, mode of delivery, or place of delivery. Qualitative studies and mixed methods studies were those that included a qualitative component, either for design (i.e. ethnography, phenomenology), data collection (i.e. focus groups, interviews, observations, diaries, oral histories), or analysis (i.e. thematic analysis, framework approach, grounded theory).

A framework approach was used to inductively develop initial themes [22] and thematic synthesis [23] and was then used iteratively based on the initial thematic framework. Study assessment included the use of a validated quality appraisal tool [24]. Confidence in the findings was assessed using the GRADE-CERQual tool [25].

### Definitions

We define the postnatal period as the time between birth, including the immediate postpartum period (first 24 hours after birth), and up to six weeks (42 days) after birth [1]. This period varies cross-culturally, but usually coincides with confinement periods and other cultural practices in the 30–45 days following birth.

We define ‘routine postnatal care’ as formal service provision that is specifically designed to support, advise, inform, educate, identify those at risk and, where necessary, manage or refer women or newborns, to ensure optimal transition from childbirth to motherhood and childhood. Postnatal care can include a wide range of activities, including risk identification (assessments, screening), prevention of complications, health education and promotion (infant feeding and care, life-skills education, postpartum family planning, nutrition, vaccines, mental health support, and prevention and management of harmful practices—including smoking and alcohol—and violence) and support for families. Routine postnatal care does not typically include specialist services for comorbidities, address social needs, or the management of conditions not related to pregnancy or postpartum care, though referrals can be made for such services as a result of routine postnatal care.

### Reflexive statement

Our study team included a medical doctor, a midwife, epidemiologists, public health researchers, and librarians, all with extensive experience in the provision and study of maternal and neonatal healthcare. We began this study with anecdotal and experiential knowledge that postnatal care is very often unavailable or inadequate, with minimal emphasis on the psychosocial needs. We believed PNC to be poorly and inequitably accessible, even in high-income settings, and especially in low- and middle-income countries (LMICs), and that due to perceived or actual poor quality care, including potential fears of mistreatment, and services not being user-friendly, families may be discouraged from seeking care. Multiple members of our study team have been involved in the direct provision of postnatal care, and in developing national and international guidelines for postnatal care.

### Search strategy

The search strategy was developed with senior librarians based on the following concepts: barriers and limitations, postnatal care, and health services needs and demands. The search was limited to qualitative and mixed-methods studies (see S1 Appendix). Databases searched included MEDLINE (OVID), PubMed, CINAHL (EBSCO), EMBASE (OVID), and EBM-Reviews (OVID), as well as a search for grey literature. The search strategy covered papers published from inception through December 2019. There were no language restrictions. Hand searching was used to identify grey literature documents on the following websites: BASE (Bielefeld University Library), OpenGrey, and on the World Health Organization. Duplicates were excluded through the EndNote X9 software using a method developed by Bramer et al. [26] Inclusion and exclusion criteria are presented in Table 1.

**Table 1:** Inclusion and exclusion criteria.

**Inclusion criteria for overall study**	**Exclusion criteria for overall study**
Studies including healthy women, and/or their partners/families who were considered to be healthy in the postnatal period, and who have had a healthy newborn	Studies reporting on views/experiences of, or access to, maternity or intrapartum services generally with no specific data on postnatal care
Studies where at least some of the extractable data are women’s, and/or their partners/families, own accounts of their views and experiences of the nature of, provision of, and/or seeking of postnatal care, irrespective of parity, mode of birth, or place of birth	Women with known complications/health conditions (e.g. depression), or after severe morbidity (e.g. near-miss)
	Services for specific conditions (e.g. HIV), or high-risk populations (e.g. multiples, preterm, low birth weight, malformations)
Studies involving postnatal care experiences with or without interaction with the health system but relating to health care (home-based, community-based care, care by family members)	Specific interventions for a singular condition (e.g breastfeeding support, family planning, mental health) or postnatal education only (e.g. parenting education)
Studies from high-, middle- and low-income countries	Studies related to care of postnatal complications or intensive care for women or newborns
	Mixed-methods studies reporting qualitative data without using a recognised qualitative approach to data collection or analysis
	Case studies, conference abstracts, or unpublished PhD or Masters’ theses
	Systematic reviews (although reference lists were reviewed)
	Evaluations of context-specific intervention programs
**Additional inclusion criteria for the current analysis on women’s views on routine postnatal care**	**Additional exclusion criteria for the current analysis on women’s views on routine postnatal care**
*Inclusion*	*Exclusion*
Studies including healthy women, who have had a healthy newborn in the preceding year	Studies focused on particular groups of women and girls, such as migrants or adolescent-only studies
Studies including womens’ own accounts (not reported only through a third party)	Very low quality papers

### Study selection

We collated records into Covidence software, excluded duplicates, and screened records based on title and abstract. To check for consistency, two members of the study team independently screened the titles and abstracts against the *a priori* inclusion/exclusion criteria and excluded irrelevant records. Before assessment of the full-texts of papers, records were categorised as follows:

1. Either “general population” or sub-populations such as adolescents, migrants
2. “Women’s view’s only”, “partners and family views only”, or “women’s, and partner/family views”.
3. Either high-income (HIC) or low or middle-income (LMIC) country setting, using the 2019 World Bank Classification Scheme.

In accord with the global nature of the review, and to ensure sufficient representation of country levels especially lower income settings, we divided the studies into either HIC or LMIC for sampling. Due to the very large number of eligible papers, 40 papers (~15%) from each geographic group (HIC or LMIC) were randomly sampled at a time, and screening and extraction was conducted until it was agreed by consensus that thematic saturation was reached for each geographic group, at which point 10 additional papers were selected from each group for confirmatory analysis (if saturation was not, 20 more papers were selected for that group, until it was agreed that saturation was reached, at which point a confirmatory set was then selected). Prior to undertaking this process, it was agreed that, if no further themes were identified after confirmatory analysis, the group was considered saturated.

Extraction of data and assessment of quality was conducted for each eligible paper by study team members. Disagreements were settled by consensus among reviewers. Themes from HIC and LMIC groups were analysed together, which notations made where the specifics or manifestation of each theme different between country groups.

Study team members did not assess papers in which they were a co-author. Two of the included studies were published in a language other than English: a Brazilian study [27] was analysed by one of the study team members fluent in Portuguese and a Japanese study [28] was translated by a Japanese-speaker into English prior to analysis. All quotes included in this manuscript were translated into English by the study authors, the respective study team members, or colleagues who assisted with translation.

Papers which did not meet either the general or specific inclusion criteria upon full review were either excluded or put aside to be evaluated separately for future analysis. Studies which did not include first-hand reports of women’s experiences were excluded; studies which focused exclusively on a sub-population (e.g. young adolescent mothers) were put aside for separate subsequent analysis.

### Data extraction and analysis

Data extraction, analysis and quality appraisal proceeded concurrently and broadly followed the ‘best fit’ framework approach described by Carroll [22]. Based on previous related reviews of antenatal care [29] and intrapartum care [30] as well as a recent thematic synthesis of ‘what matters to women’ during the postnatal period [31] we used a deductive approach to develop a thematic framework comprising four broad concepts (Resources and access; Behaviours and attitudes; External influences; What women want and need) as well as a number of sub-themes (see S2 Appendix). We then used thematic synthesis techniques [23] to confirm our *a priori* framework, or to develop new themes where emerging data failed to fit. We began by using an Excel spreadsheet to record pertinent details from each study (e.g. author, country, publication date, study design, setting and location of birth, setting and location of postnatal care, sample size, data collection methods, participant demographics, contexts, study objectives). The four concepts from our *a priori* framework were added to the Excel sheet and the author-identified findings from each study were extracted (along with supporting quotes) and mapped to the framework as appropriate. Any codes which did not map to the framework were placed in a section marked ‘other’ to allow for the emergence of new sub-themes or concepts. This process included looking for what was similar between papers and for what contradicted (‘disconfirms’) the emerging themes. For the disconfirming process we consciously looked for data that would contradict our emerging themes, or our prior beliefs, and views related to the topic of the review.

### Quality assessment

Included studies were appraised using an instrument developed by Walsh and Downe [32] and modified by Downe et al. [33]. Studies were rated against 11 pre-defined criteria [33], and then allocated a score from A–D (including + and -), where A+ was the highest and D- the lowest (see Table 2). Studies rated with a D were excluded from further data analysis.

**Table 2:** Ratings for quality assessments of studies.

A represented a study with no, or few flaws, with high credibility, transferability, dependability and confirmability.
B, a study with some flaws, unlikely to affect the credibility, transferability, dependability and/or confirmability of the study.
C, a study with some flaws that may affect the credibility, transferability, dependability and/or confirmability of the study.
D, a study with significant flaws that are very likely to affect the credibility, transferability, dependability and/or confirmability of the study.

Studies were appraised by each reviewer independently and a 10% sample was cross-checked by a different study team member to ensure consistency. Each reviewer was asked to extract and assess both LMIC and HIC papers in order to increase intra-rater reliability between the two geographic groups. Any studies where there were scoring discrepancies of more than a grade were referred to another study team member for moderation.

Once the framework of descriptive themes (or review findings) was agreed by the study team, the level of confidence in each review finding was assessed using the GRADE-CERQual tool [34] and agreed by consensus between two study team members. GRADE-CERQual assesses the methodological limitations and relevance to the review of the studies contributing to a review finding, the coherence of the review finding, and the adequacy of data supporting a review finding. Based on these criteria, review findings were graded for confidence using a classification system ranging from ‘high’ to ‘moderate’ to ‘low’ to ‘very low’. Following CERQual assessment the review findings were grouped into higher order analytical themes and the final framework was agreed by consensus amongst the study team.

## Results

### Papers included in overall study and analytic sample

**Fig 1:**
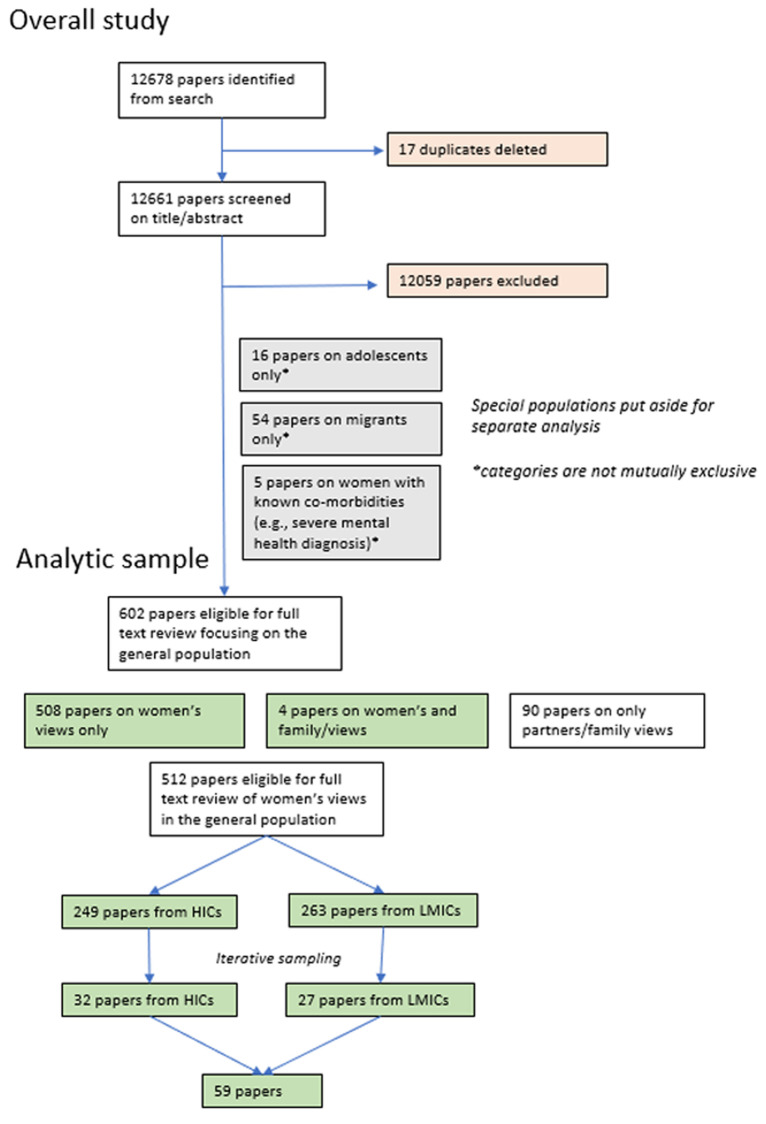
Flow diagram of included papers.

Our systematic searches yielded 12,678 records, of which 17 were duplicates. An additional 12,149 were excluded by title and by abstract, leaving 602 for full text review (See Fig 1).

Our final list of articles for the analytic sample included 59 studies with views from women in the general population on routine postnatal care, with 32 coming from HICs and 27 from LMICs. Specifically, of the LMIC studies, 6 were from low income countries, 12 from lower-middle income countries, and 9 from upper-middle income countries. The global representation of studies was reasonably wide with 17 coming from Europe, 13 from Africa, 10 from North America, 9 from Asia, 4 from the Middle East, 4 from Australasia, and 2 from South America. The two South American studies were both from Brazil and, although we actively searched our entire database for studies from other Latin American countries, no others fulfilled our inclusion criteria. The studies were generally of good quality with an average quality rating of B and were mainly qualitative and descriptive in design. A full list of the included studies with relevant characteristics is shown in Table 3.

**Table 3:** Characteristics of included studies, alphabetical by study first author.

Author(s) and Study	Year	Country and income level	Setting (urban/rural) health facility/community/home	Research Design	Participants	Quality rating
Abushaikha L, Khalaf I. *Exploring the Roles of Family Members in Women’s Decision to Use Postpartum Healthcare Services from the Perspectives of Women and Health Care Providers* [35]	2014	Jordan (Upper middle)	Urban	Qualitative and descriptive	24 women in 3 focus groups	C
			Community based			
Alyahya MS, Khader YS, Batieha A, Asad M. *The quality of maternal-fetal and newborn care services in Jordan*: *a qualitative focus group study* [36]	2019	Jordan (Upper middle)	Unclear	Qualitative and descriptive	52 women in 12 focus groups	B
			Facility based			
Aston M, Price S, Monaghan J, Sim M, Hunter A, Little V. *Navigating and negotiating information and support*: *Experiences of first-time mothers* [37]	2018	Canada (High)	Urban and rural.	Qualitative and analysed using feminist theory and discourse analysis	19 Self-identified first-time mothers within 12 months of birth/adoption	A
			Community based			
Aune I, Dahlberg U, Ingebrigtsen O. *Parents’ experiences of midwifery students providing continuity of care* [38]	2012	Norway (High)	Urban.	Qualitative and analysed using systematic text condensation	8 women and 5 men (partners)	B-
			Community and home			
Ayanore MA, Pavlova M, Biesma R, Groot W. *Stakeholders’ views on maternity care shortcomings in rural Ghana*: *An ethnographic study among women*, *providers*, *public*, *and quasiprivate policy sector actors* [39]	2017	Ghana (Lower middle)	Rural.	Qualitative using ethnographic approach	90 women in 9 focus groups plus interviews with providers and policy actors	C+
			Facility and community			
Baker SR, Choi PYL, Henshaw CA, Tree J. *I felt as though I’d been in jail’*: *women’s experiences of maternity care during labour*, *delivery and the immediate postpartum* [40]	2005	United Kingdom (High)	Unclear.	Qualitative and descriptive	24 primiparous women	B
			Facility based			
Beake S, McCourt C, Bick D. *Women’s views of hospital and community-based postnatal care*: *the good*, *the bad and the indifferent* [41]	2005	United Kingdom (High)	Urban.	Qualitative and descriptive	22 women	B
			Facility and community			
Bhattacharyya S, Issac A, Rajbangshi P, Srivastava A, Avan BI. *“Neither we are satisfied nor they”-users and provider’s perspective*: *a qualitative study of maternity care in secondary level public health facilities*, *Uttar Pradesh*, *India* [42]	2015	India (Lower middle)	Unclear.	Qualitative and descriptive	24 women	A
			Facility based			
Cronin C, & McCarthy G. *First-time mothers—identifying their needs*, *perceptions and experiences* [43]	2003	Ireland (High)	Urban.	Qualitative and descriptive	13 women	C
			Facility based			
Dahlberg U, Haugan G, Aune I. Women’s experiences of home visits in the early post-natal period [44]	2016	Norway (High)	Urban.	Qualitative and analysed using systematic text condensation	24 women in 6 focus groups	B-
			Home based			
Diamond-Smith N, Thet MM, Khaing EE, Sudhinaraset M. *Delivery and postpartum practices among new mothers in Laputta*, *Myanmar*: *intersecting traditional and modern practices and beliefs* [45]	2016	Myanmar (Lower middle)	Urban and rural.	Qualitative using grounded theory approach	24 women (plus 10 male partners and 10 grandmothers)	B+
			Home based			
Forster DA, McLachlan HL, Rayner J, Yelland J, Gold L, Rayner S. *The early postnatal period*: *exploring women’s views*, *expectations and experiences of care using focus groups in Victoria*, *Australia* [46]	2008	Australia (High)	Urban and rural.	Qualitative and descriptive	50 women (and 2 male partners)	B
			Facility and home			
Frei IA, & Mander R. *The relationship between first-time mothers and care providers in the early postnatal phase*: *an ethnographic study in a Swiss postnatal unit* [47]	2011	Switzer-land (High)	Unclear.	Qualitative using ethnographic approach	10 primiparous women	A
			Facility based			
Gaboury J, Capaday S, Somera J, Purden M. *Effect of the Postpartum Hospital Environment on the Attainment of Mothers’ and Fathers’ Goals* [48]	2017	Canada (High)	Unclear.	Qualitative and descriptive	10 women (and 8 male partners)	A
			Facility based			
Gupta ML, Aborigo RA, Adongo PB, Rominski S, Hodgson A, Engmann CM, Moyer CA. *Grandmothers as gatekeepers*? *The role of grandmothers in influencing health-seeking for mothers and newborns in rural northern Ghana* [49]	2015	Ghana (Lower middle)	Rural.	Qualitative and descriptive	72 interviews including 35 with women plus 8 Focus groups with 81 grandmothers	C+
			Facility, community and home			
George L. *Lack of Preparedness*: *Experiences of First-Time Mothers* [50]	2005	USA (High)	Urban.	Qualitative using grounded theory approach	10 primiparous women	B
			Home based			
Henderson V, Stumbras K, Caskey R, Haider S, Rankin K, Handler A. *Understanding Factors Associated with Postpartum Visit Attendance and Contraception Choices*: *Listening to Low-Income Postpartum Women and Health Care Providers* [51]	2016	USA (High)	Urban.	Qualitative and descriptive	20 mothers and 12 healthcare providers	A
			Facility based			
Hindley, J. *Having a baby in Balsall Heath*: *women’s experiences and views of continuity and discontinuity of midwifery care in the mother-midwife relationship*: *a review of the findings from a report of a research project commissioned by ’Including Women’* [52]	2005	UK (High)	Urban.	Qualitative and descriptive	20 mothers	B
			Facility, community and home			
Hoang H, Le Q, Terry D. *Women’s access needs in maternity care in rural Tasmania*, *Australia*: *a mixed methods study* [53]	2014	Australia (High)	Rural.	Mixed methods using a survey and semi-structured interviews	210 women completed the survey and 22 mothers participated in the interviews	B
			Community			
Humbert L, Roberts TL. *The Value of a Learner’s Stance*: *Lessons Learned from Pregnant and Parenting Women* [54]	2009	USA (High)	Urban.	Qualitative and descriptive	24 focus groups with 143 women (aged 14–45)–all receiving Medicaid	C+
			Facility			
Izugbara CO, Wekesah F. *What does quality maternity care mean in a context of medical pluralism*? *Perspectives of women in Nigeria* [55]	2018	Nigeria (Lower middle)	Urban, Semi-urban and rural.	Qualitative and descriptive	173 women in total: 16 focus groups with 130 women and 43 interviews	C+
			Facility and community			
Kanengoni B, Andajani-Sutjahjo S, Holroyd E. *Women’s experiences of disrespective and abusive maternal healthcare in a low resource rural setting in eastern Zimbabwe* [56]	2019	Zimbabwe (Lower middle)	Rural.	Qualitative and descriptive	20 women, 8 participated in interviews and another 12 in 2 focus groups	C
			Facility based			
Khalaf IA. *Jordanian women’s perceptions of post-partum health care* [57]	2007	Jordan (Upper middle)	Unclear.	Qualitative and descriptive	24 women in 3 focus groups	C+
			Facility based			
Kirca N & Ozcan S. *Problems Experienced by Puerperants in the Postpartum Period and Views of the Puerperants about Solution Recommendations for these Problems*: *A Qualitative Research* [58]	2018	Turkey (High)	Urban.	Qualitative and descriptive	24 interviews with women	C
			Facility and community			
Kurth E, Krähenbühl K, Eicher M, Rodmann S, Fölmli L, Conzelmann C, Zemp E. *Safe start at home*: *what parents of newborns need after early discharge from hospital–a focus group study* [59]	2016	Switzerland (High)	Urban and rural.	Qualitative using a ‘playful’ design (creating symbolic structures and images with plastic bricks)	24 participants in 6 focus groups including 20 women and 4 male partners	A-
			Facility and community			
Kurth E, Spichiger E, Zemp Stutz E, Biedermann J, Hösli I, Kennedy HP. *Crying babies*, *tired mothers—challenges of the postnatal hospital stay*: *an interpretive phenomenological study* [60]	2010	Switzerland (High)	Urban.	Qualitative using interpretive phenomenology	15 women of diverse parity and educational backgrounds	A-
			Facility based			
Leirbakk MJ, Torper J, Engebretsen E, Opsahl J N, Zeanah P, Magnus JH. *Formative research in the development of a salutogenic early intervention home visiting program integrated in public child health service in a multi-ethnic population in Norway* [61]	2018	Norway (High)	Urban.	Qualitative using a formative approach utilizing data from multiple sources	18 women in 5 focus groups	B
			Community and home			
Lewis L. *Postnatal clinics*: *Midwives and women’s experiences* [62]	2009	UK (High)	Urban.	Qualitative and descriptive	8 postnatal women (and 6 community midwives)	B
			Community based			
McCarter D, Macleod CE. *What Do Women Want*? *Looking Beyond Patient Satisfaction* [63]	2016	USA (High)	Urban.	Qualitative and descriptive	20 women of various parities (including several first-time mothers) plus 2 couples expecting their first child	C+
			Facility based			
Memon Z, Zaidi S, Riaz A. *Residual Barriers for Utilization of Maternal and Child Health Services*: *Community Perceptions From Rural Pakistan* [64]	2015	Pakistan (Lower middle)	Rural.	Qualitative and exploratory	Total number not provided. 20 focus groups with an average of 10 women with children under age of 5 plus fathers of children under 5 and community health workers	C
			Facility based			
Miteniece E, Pavlova M, Shengelia L, Rechel B, Groot W. *Barriers to accessing adequate maternal care in Georgia*: *a qualitative study* [65]	2018	Georgia (Upper middle)	Urban and rural.	Qualitative and exploratory	60 women, nulliparous and multiparous, aged 19–42 plus providers and policy-makers	A
			Facility based			
Morris JL, Short S, Robson L, Andriatsihosena MS. *Maternal Health Practices*, *Beliefs and Traditions in Southeast Madagascar* [66]	2014	Madaga-scar (Low income)	Urban.	Mixed methods including questionnaires, interviews and focus groups.	629 in total, 256 in qualitative part of the study, including 60 women in 6 FGD, and interviews with 10 women with children less than 1year old	C
			Community and home			
Mrisho M. Obrist B, Schellenberg JA, Haws RA, Mushi AK, Mshinda H, Tanner M. *Schellenberg D*. *The use of antenatal and postnatal care*: *perspectives and experiences of women and health care providers in rural southern Tanzania* [67]	2009	Tanzania (Lower middle)	Rural.	Qualitative and descriptive	74 women and healthcare workers in total. Of the total 58 were women and of these 39 had a young child less than one year old and 19 were pregnant	B-
			Community based			
Mrisho M, Schellenberg JA, Mushi AK, Obrist B, Mshinda H, Tanner M, Schellenberg D. *Understanding home-based neonatal care practice in rural southern Tanzania* [68]	2008	Tanzania (Lower middle)	Rural.	Qualitative and descriptive	A total of 40 in-depth interviews (5 in 8 villages) and 16 focus groups (5 per village). Interviews were with postnatal women, pregnant women and 8 TBA’s. Focus groups (2 per village) with 6–8 women who had given birth at least once	C
			Facility and home			
Munday R. *Women’s experiences of the postnatal period following a planned homebirth*: *A phenomenological study* [69]	2003(a)	Canada (High)	Unclear.	Qualitative using interpretive phenomenology	10 women who planned a homebirth	C+
			Home based			
Munday R. *Women’s experiences of the postnatal period following a planned homebirth*: *A phenomenological study* [70]	2003(b)	Canada (High)	Unclear.	Qualitative using interpretive phenomenology	10 women who planned a homebirth	C+
			Home based			
Nakano AMS, Beleza AC, Gomes FA, Mamede FV. *O cuidado no "resguardo"*: *as vivências de crenças e tabus por um grupo de puérpera* [27].	2003	Brazil (Upper middle)	Urban.	Qualitative and descriptive	20 women	C-
			Home based			
Newbrander W, Natiq K, Shahim S, Hamid N, Skena NB. *Barriers to appropriate care for mothers and infants during the perinatal period in rural Afghanistan*: *a qualitative assessment* [71]	2014	Afghani-stan (Low)	Rural.	Qualitative and descriptive	30 interviews with women plus 29 focus groups and 15 direct observations	B-
			Home based			
Noguchi M, Takahashi N, Fujita W, Asaka Y, Takamuro N. *Perceptions of women who utilized public postpartum health care services in Sapporo City*, *Japan* [72]	2018	Japan (High)	Urban.	Mixed methods using a survey and in-depth interviews	21 interviews with mothers	C
			Community based			
Persson EK, Fridlund B, Kvist LJ, Dykes AK. *Mothers’ sense of security in the first postnatal week*: *interview study* [73]	2011	Sweden (High)	Urban.	Qualitative and descriptive	10 women in 3 focus groups plus 4 individual interviews (14 women in total)	A
			Facility based			
Probandari A, Arcita A, Kothijah K, Pamungkasari EP. *Barriers to utilization of postnatal care at village level in Klaten district*, *central Java Province*, *Indonesia* [74]	2017	Indonesia (Upper middle)	Rural.	Qualitative and descriptive	8 mothers with postnatal complications (plus 6 family members)	B
			Facility and home based			
Puthussery S, Twamley K, Macfarlane A, Harding S, Baron M. *‘You need that loving tender care’*: *maternity care experiences and expectations of ethnic minority women born in the United Kingdom* [75]	2010	UK (High)	Urban.	Qualitative and descriptive	34 mothers of Black Caribbean, Black African, Indian, Pakistani, Bangladeshi and Irish descent	B-
			Facility based			
Raven JH, Chen Q, Tolhurst RJ, Garner P. *Traditional beliefs and practices in the postpartum period in Fujian Province*, *China*: *a qualitative study* [76]	2007	China (Upper middle)	Urban and rural.	Qualitative and descriptive	12 mothers (plus 12 fathers & 12 grandmothers)	B
			Home based			
Razurel C, Bruchon-Schweitzer M, Dupanloup A, Irion O, Epiney M. *Stressful events*, *social support and coping strategies of primiparous women during the postpartum period*: *a qualitative study* [77]	2011	Switzer-land (High)	Urban.	Qualitative and descriptive	60 first-time mothers	B+
			Facility, community and home			
Ribeiro JP, da Costa de Lima FB, da Silva Soares TM, Oliveira BB, Klemtz FV, Lopes KB, Hartmann M. *Needs Felt by Women in the Puerperal Period* [78]	2019	Brazil (Upper middle)	Urban.	Qualitative and descriptive	20 mothers (10 in the immediate postpartum and 10 in the ‘remote’ postpartum phase)	C+
			Facility based			
Rodin D, Silow-Carroll S, Cross-Barnet C, Courtot B, Hill I. *Strategies to Promote Postpartum Visit Attendance Among Medicaid Participants* [79]	2019	USA (High)	Mixed.	Analysis of qualitative case studies from various data sources	Coded notes from qualitative case studies including 739 interviews with 1,074 key informants and 133 focus groups with 951 women	C+
			Facility and community based			
Rouhi M, Stirling CM, Crisp EP. *Mothers’ views of health problems in the 12 months after childbirth*: *A concept mapping study* [80]	2019	Australia (High)	Urban.	Mixed methods–Concept mapping study	81 mothers	B-
			Unclear			
Sacks E, Moss WJ, Winch PJ, Thuma P, van Dijk JH, Mullany LC. *Skin*, *thermal and umbilical cord care practices for neonates in southern*, *rural Zambia*: *a qualitative study* [81]	2015	Zambia (Lower middle)	Rural.	Qualitative and descriptive utilizing interviews, focus groups and observations	36 interviews (24 with mothers) and 39 participants at 5 Focus groups (including some TBAs)	A
			Home and facility			
Shaban IA, Al-Awamreha K, Mohammadb K, Gharaibehb H. *Postnatal women’s perspectives on the feasibility of introducing postpartum home visits*: *a Jordanian study* [82]	2018	Jordan (Upper middle)	Unclear.	Qualitative and descriptive	30 women with healthy newborns (vaginal or cesarean birth), 17–43 years old, primiparous and multiparous	B
			Home based			
Sharkey A, Yansaneh A, Bangura PS, Kabano A, Brady E, Yumkella F, Diaz T. *Maternal and newborn care practices in Sierra Leone*: *a mixed methods study of four underserved districts* [83]	2017	Sierra Leone (Low)	Rural.	Mixed methods utilizing survey data, interviews and focus groups	98 Interviews and 15 Focus Groups (8 Focus groups made up of men only)	B-
			Community based			
Sialubanje C, Massar K, Hamer DH, Ruiter RAC. *Understanding the psychosocial and environmental factors and barriers affecting utilization of maternal healthcare services in Kalomo*, *Zambia*: *a qualitative study* [84]	2014	Zambia (Lower middle)	Urban and rural.	Qualitative and descriptive	141 women in 12 Focus groups, 12 women per FDG except one with 9 women; plus 35 in-depth interviews with key informants	B
			Facility based			
Tesfaye G, Chojenta K, Smith R, Loxton D. *Delaying Factors for Maternal Health Service Utilization in Eastern Ethiopia*: *A Qualitative Exploratory Study* [85]	2019	Ethiopia (Low)	Urban and rural.	Qualitative and exploratory	20 women (plus 19 mothers-in-law, 13 TBAs, 24 husbands, 12 health extension officers).	B
			Community based			
Tully KP, Steube AM, Verbiest SB. *The fourth trimester*: *a critical transition period with unmet maternal health needs* [86]	2017	USA (High)	Urban.	Type of delphi study utilizing data from various sources and interviews with key stakeholders	18 mostly immigrant mothers from underprivileged urban areas	C
			Facility and community			
Waiswa P, Kemigisa M, Kiguli J, Naikoba S, Pariyo GW, Peterson S. *Acceptability of evidence-based neonatal care practices in rural Uganda–implications for programming* [87]	2008	Uganda (Low)	Rural.	Qualitative and descriptive	2 focus groups with young mothers, 4 with older mothers (> 30 yrs), 2 focus groups with fathers, 2 with child minders (older children up to 13 yrs)	C+
			Facility, community and home			
White PM. *Crossing the river*: *Khmer women’s perceptions of pregnancy and postpartum* [88]	2002	Cambodia (Lower middle)	Rural and urban.	Qualitative and descriptive incorporating ethnographic approaches	88 participants total, including mothers, TBAs, and midwives (41 interviews and 11 focus groups)	B-
			Community based			
Woodward BM, Zadoroznyj M, Benoit C. *Beyond birth*: *Women’s concerns about post-birth care in an Australian urban community* [89]	2016	Australia (High)	Urban.	Qualitative and descriptive	15 mothers who had given birth in different environments	B
			Facility, community and home			
Yeh YC, St John W, Chuang YH, Huang YP. T*he care needs of postpartum women taking their first time of doing the month*: *a qualitative study* [90]	2017	Taiwan (High)	Urban.	Qualitative and descriptive utilizing interviews	27 new mothers aged 25–39 interviewed in PNC facility	B
			PNC Nursing facility			
Young E. *Maternal Expectations*: *Do they match experience*? [91]	2008	UK (High)	Urban.	Qualitative and descriptive utilizing focus groups and interviews	11 interviews with 1^st^ time mothers aged 24–30 with a range of EPDS depression scale scores	C
			Facility based			
Zamawe C, Masache GC, Dube AN. *The role of the parents’ perception of the postpartum period and knowledge of maternal mortality in uptake of postnatal care*: *a qualitative exploration in Malawi* [92]	2015	Malawi (Low)	Rural.	Qualitative and descriptive utilizing focus groups	36 women in three focus groups, 14 men in 1 focus group. All married farmers. Women 18–25 years, men between ages of 25 and 35 years. >50% finished primary education	C
			Facility based			

TBA: traditional birth attendant.

### Findings

This process generated 20 review findings. Following discussions amongst the study team, these descriptive themes were then mapped against our *a priori* framework themes to generate our final analytical themes. *Resources and Access* was split into two separate themes: *Access and Availability* and *Physical and Human Resources*. We changed *Behaviours and Attitudes* to *Social Norms* to better reflect the larger group of stakeholders influencing maternal choice or behaviour, and we changed the title of *What Women Want and Need* to *Experience of Care* to better reflect the experiential nature of the findings.

Our analysis reinforced some aspects of the themes in our *a priori* framework and modified or expanded others. This final framework includes twenty-one themes and five overarching study findings: *Access and Availability; Physical and Human Resources; External Influences; Social Norms; and Experience of Care*. Our final framework displaying the analytical themes and descriptive themes, with their associated CERQual gradings, is shown in Table 4.

**Table 4:** Review findings and CERQual gradings.

Analytical Theme	Review finding	Contributing papers	Supporting Quote	CERQual Grade
**ACCESS & AVAILABILITY**	**1. Flexible contact opportunities—**Women in included studies expressed a preference for a wider range of access options as well as greater flexibility in provider managed appointment systems. The provision of home visits was valued by these women in a number of different contexts and the availability of drop-in clinics, out of hours services, and telephone or online services were also highlighted as examples of more user-friendly systems.	13 studies (7 HIC; 6 LMIC): Ayanore 2017 (Ghana); Frei 2011 (Switzerland); Henderson 2016 (USA); Hindley 2005 (UK); Hoang 2014 (Australia); Khalaf 2007 (Jordan); Kurth 2010 (Switzerland); Leirbakk 2018 (Norway) Lewis 2009 (UK); Mrisho, 2009 (Tanzania); Ribiero 2019 (Brazil); Shaban 2018 (Jordan); Zamawe 2015 (Malawi)	"I feel that the schedule for the visit must be flexible according to each mother, and the schedule should be determined by the mother and her health care provider together". [Shaban 2018, Jordan]	Moderate
			"I guess if there was someone in a situation where they didn’t have the support, it was just a single mom and their child, I guess if someone can come into their home or something, that would probably be really convenient.” [Henderson 2016, USA]	
	**2. Personal resources:** In the included studies, additional costs associated with transport to and from health facilities, payment for childcare, healthcare insurance payments, the potential costs of medicines for mother or newborn as well as the loss of household income associated with clinic attendance all acted as barriers to PNC engagement.	13 studies (3 HIC, 10 LMIC): Ayanore 2017 (Ghana); Bhattacharyya, 2015 (India); Gupta 2015 (Ghana); Hoang, 2014 (Australia); Miteniece 2018 (Georgia); Mrisho 2008 (Tanzania); Mrisho 2009 (Tanzania); Newbrander 2014 (Afghanistan); Noguchi 2018 (Japan); Rodin 2019 (USA); Shaban 2018 (Jordan); Sialubanje 2014 (Zambia); Zamaw, 2015 (Malawi)	"The clinic is far away, so we cannot get there by walking. We cannot rent a car to go because there is not enough money, not even 5 Afs [US$ 0.10], in my husband’s pocket”. [Newbrander 2014, Afghanistan]	Moderate
			"The cost of travel was pretty expensive and also trying to organise for someone to look after my other son because he doesn’t travel well". [Hoang 2014, Australia]	
	**3. Proximity of health facility**: For some women in the included studies, the convenience of having a health facility close to where they lived encouraged attendance for postnatal care, but for women living in predominantly rural areas inadequate transport networks and/or poor infrastructure acted as a barrier to access.	9 studies (1 HIC, 8 LMIC): Ayanore, 2017 (Ghana); Gupta 2015, (Ghana); Hoang 2014 (Australia); Miteniece 2018 (Georgia); Mrisho 2009 (Tanzania); Newbrander 2014 (Afghanistan); Tesfaye 2019 (Ethiopia): Waiswa, 2008 (Uganda); Zamawe 2015 (Malawi)	“Distance is an issue for women from rural areas, because in the capital the care is more adequate and modern than in rural areas.” [Miteniece 2018, Georgia]	Low
	**4. Value of women’s time**—In a number of settings, the amount of time women had to wait at a health facility to see a healthcare provider for a postnatal check-up (regardless of whether they had a timed appointment or not) was too long and often led to frustration and/or additional expense. In some instances, this was compounded by cursory exchanges with healthcare providers, leaving women feeling undervalued and frustrated.	8 studies (2 HIC, 6 LMIC): Ayanore 2017 (Ghana); Alyahaya, 2019 (Jordan); Hindley, 2005 (UK); Humbert, 2009 (USA); Kanengoni 2019 (Zimbabwe); Newbrander 2018 (Afghanistan); Probandari 2017 (Indonesia); Zamawe 2015 (Malawi)	"The problem with the clinic was that they would make us wait for a very long time in the queue without being served. They opened at 8 in the mornings and I am only leaving at 3pm after being served because there are no midwives there" [Kanengoni 2019, Zimbabwe]	Low
**PHYSICAL AND HUMAN RESOURCES**	**5. Physical resources at facilities—**Women in all settings represented by the included studies perceived a deficit, reporting that some health facilities were under-resourced and, especially in LMICs, also expressed the unavailability of drugs or equipment and inconsistencies in the supply of water or electricity to the health facility.	6 studies (2 HIC, 4 LMIC): Bhattacharyya 2015 (India); Forster 2008 (Australia); Izugbara 2018(Nigeria); Mrisho 2009, (Tanzania); Puthussery 2010 (UK); Waiswa 2008, (Uganda)	“They (sweeper) clean the ward and toilet once a day. But the toilet is dirty most of the time. Sometimes, women do not pour enough water. Water supply is also not regular. " [Bhattacharyya, India, 2015]	Low
			“So they moved me to another ward, not another ward, just another area of the ward, but there was no light. And they gave me like a pen like yours… a pen-torch, and that’s what I needed to use to see my baby to feed my baby”. [Puthussery 2015, UK]	
	**6. Human resources at facilities–**Many women in the included studies reported that health facilities were under-resourced in terms of staff resulting in limited time for staff to spend with patients	9 studies (5 HIC, 4 LMIC): Ayanore 2017, (Ghana); Baker 2005, (UK); Beake 2007, (UK); Hindley 2005 (UK); Miteniece 2018, (Georgia); Mrisho 2009 (Tanzania); Probandari 2017(Indonesia); Puthussery 2010(UK); Tully 2017 (USA)	"You just feel that there was so few midwives to look after so many women [on the postnatal ward] that they just couldn’t give you the… you know the time that you wanted… . " [Hindley 2005, UK 2 UK quotes]	Low
			“Those who go for weight monitoring spend less time at the clinic than those who go for vaccination. This is because there is one health care provider; we suggest that there is a need to increase the number of health care providers” [Mrisho 2009, Tanzania]	
**EXTERNAL INFLUENCES**	**7. Importance of environment—**For women in the studies who gave birth in health facilities the unhygienic, dirty and dilapidated conditions in some postnatal wards made them feel disappointed, depressed and occasionally unsafe. For others, not being able to control the often noisy and disruptive atmosphere on the postnatal ward generated feelings of frustration and despair, prompting a few to leave earlier than planned. For women who gave birth at home, the relatively calm and familiar home environment enabled them to feel more relaxed and in control of their immediate postnatal experiences.	10 studies (9 HIC, 1 LMIC): Beake, 2005 (UK); Bhattacharyya, 2015 (India); Cronin, 2003 (Ireland); Forster, 2008 (Australia), Frei, 2011 (Switzerland); Gaboury, 2017 (Canada); Munday, 2003a (UK); Noguchi, 2018 (Japan); Puthussery, 2010 (UK); Woodward, 2016 (Australia).	“I would have liked the whole thing to have happened in a much nicer environment. [Name of the Maternity unit] is grim to say the least, it’s Victorian-looking, it’s grey, it’s dark and dull and, having said that, the staff, the midwives, were fine, were great…but the place itself was quite a depressing place to give birth in and a bit frightening really” [Puthussery 2010, UK]	Moderate
			*Facility birth*: "I did not have one minute to myself. People were just coming in all the time’ and if it’s not your visitor, it’s the girl across the way" [Cronin 2003, Ireland].	
			*Homebirth*: "You can go and sit in your witches brew (herbal sits bath) and not bother about anybody…it’s nice just being able to pad around in the nude if you want to" [Munday 2003b, UK]	
	**8. Influence of traditional practices**—In included studies from LMIC contexts, women preferred to observe traditional (or cultural) practices relating to postnatal care. For some women this incorporated a variety of practices (sometimes involving seclusion or isolation) frequently associated with ’doing the month’; for others it involved the use of medicines, herbs and spiritual purification under the guidance of a healer or TBA; and for others the preference to be seen by a local healer or TBA was born out of trust, convenience and/or economic necessity. In a few settings women felt conflicted between observing traditional practices (advocated by influential family members) and the approaches recommended by healthcare providers.	16 studies (2 HIC, 14 LMIC): Diamond-Smith, 2016 (Myanmar); Gupta, 2015 (Ghana); Humbert, 2009 (USA); Izugbara, 2018 (Nigeria); Memon, 2016 (Pakistan); Morris, 2014 (Madagascar); Mrisho, 2008 (Tanzania); Nakano, 2003 (Brazil); Newbrander, 2014 (Afghanistan); Probandari, 2017 (Indonesia); Raven, 2007 (China); Sacks, 2015 (Zambia); Sharkey, 2017 (Sierra Leone); Tesfaye, 2019 (Ethiopia); Yeh, 2017 (Taiwan); White, 2002 (Cambodia).	"… The families do not want the women to go out of home before two months of birth for fear of the evil eye” [Tesfaye 2019, Ethiopia]	Moderate
			"Old family members and friends tell us to have traditional food during this period. We follow their advice because we don’t know what to do in this period. But if we do follow this diet we still don’t know if we will have some problems. The doctor gave us some suggestions, but our parents promoted the traditional way. It is difficult to make a choice" [Raven 2007, China]	
	**9. Women’s autonomy**–In the included studies, women’s decisions to engage with formal postnatal services was often influenced by key family members and/or societal norms relating to women’s autonomy and/or their ability to travel independently. In some settings, the role of maternity care decision-maker was taken by the mother-in-law, sometimes with additional influence by the husband. Women’s capacity to engage with postnatal care was largely dependent on the value these family members placed on postnatal services.	10 studies (10 LMIC): Abushaikha, 2014 (Jordan); Ayanore, 2017 (Ghana); Diamond-Smith, 2016 (Myanmar); Gupta, 2015 (Ghana); Kirca, 2018 (Turkey); Mrisho, 2009 (Tanzania); Newbrander, 2014 (Afghanistan); Raven, 2007 (China); Tesfaye, 2019 (Ethiopia); Waiswa, 2008 (Uganda)	“Mothers-in-law say, ‘We stayed indoors and did not go to doctors for our problems, so you should not go to doctors’.” [Newbrander 2014, Afghanistan]	Moderate
			"M: How would you know if your baby is sick?	
			R: I wouldn’t know unless I ask my mother-in-law.	
			M: How about if it is a convulsion?	
			R: We will go to clinic but with my mother in-law’s permission" [Gupta 2015, Ghana]	
	**10. Privacy**–In some studies from HIC settings, women’s need for privacy was sometimes expressed in terms of their inability to engage in confidential conversations with healthcare providers because of a lack of space or a lack of sensitivity. In other settings the relatively open nature of shared postnatal wards left women feeling exposed and vulnerable at a time when they felt most in need of privacy. For studies on women who gave birth at home in HIC contexts, the ability to control their environment to regulate visitor access and periods of rest was viewed in positive terms.	8 studies (6 HIC, 2 LMIC): Bhattacharyya 2015 (India); Beake, 2005 (UK); Gaboury 2017 (Canada); Humbert 2009 (USA); Khalaf, 2007 (Jordan); Kurth 2010 (Switzerland); Munday 2003b (Canada); Woodward 2006 (Australia)	“I would not feel comfortable breastfeeding in a shared room with a curtain” [Gaboury 2017, Canada]	Low
			“I’m a private person anyway and I want to be enclosed and everyone was yanking back the curtains all the time, which I think was a bit annoying” [Beake 2005, UK]	
	**11. Influence of private PNC provision**—In a few settings in the included studies, women with access to financial resources chose to make use of private healthcare facilities because they perceived maternity care to be of higher quality. In some instances, this perception proved to be unfounded and women were left feeling disappointed by the lack of postnatal care on offer. In other instances, women were forced to pay for private treatment because the public hospital did not have the resources to provide the required level of postnatal care	7 studies (2 HIC, 5 LMIC): Alyahaya, 2019 (Jordan); Forster 2008 (Australia); Izugbara 2018 (Nigeria); Khalaf 2007 (Jordan); Memon 2016 (Pakistan); Probandari 2017 (Indonesia); Woodward 2016 (Australia)	"One of the reasons we went private was because it was a longer stay and we didn’t feel like two nights was adequate preparation to learn to take care of a child… but I could imagine that with your second child you might want to stay shorter in total” [Forster 2008, Australia]	Very Low
**SOCIAL NORMS**	**12. Value of formal postnatal care**—In studies from some LMIC settings, women did not recognise the need for formal postnatal services and only visited health facilities when they or their infant became unwell or experienced complications. Occasionally these priorities were exacerbated by healthcare providers who either failed to promote postnatal care practices or devalued the services they offered.	13 studies (13 LMIC): Abushaikha, 2014 (Jordan); Ayanore, 2017 (Ghana); Alyahaya, 2019 (Jordan); Khalaf, 2007 (Jordan); Memon, 2015 (Pakistan); Mrisho, 2008 (Tanzania); Mrisho, 2009 (Tanzania); Probandari, 2017 (Indonesia); Shaban, 2018 (Jordan) Sialubanje, 2014 (Zambia); Tesfaye, 2019 (Ethiopia); Waiswa, 2008 (Uganda); Zamawe, 2015 (Malawi)	"I have given birth to my first child at home and didn’t visit a health facility for check-up and nothing happened to the child. So, I don’t want to waste my time by going there" [Tesfaye 2019, Ethiopia].	Moderate
			"I had visited the health centre only once during the postpartum period because when I went they told me there was no need to visit the health centre when I was not complaining of anything. So why visit the health centre?" [Khalaf 2007, Jordan]	
	**13. Trust in the system**–In the included studies, women’s willingness to engage with PNC services was sometimes undermined by a lack of trust in the system. Women expressed this issue in a number of ways including the need to provide informal payments or gifts/bribes to ensure quality of care, a lack of faith in the clinical skills of the provider, the belief that personal information would not remain confidential and the perception that disclosure of a mental health issue (like postnatal depression) might lead to their infant being taken away.	12 studies (1 HIC, 11 LMIC): Alyahaya 2019 (Jordan); Ayanore 2017 (Ghana); Izugbara 2018 (Nigeria); Kanengoni 2019 (Zimbabwe); Khalaf 2007 (Jordan); Newbrander 2014 (Afghanistan); Probandari 2017 (Indonesia); Shaban 2018 (Jordan); Sialubanje, 2014 (Zambia); Tully 2017 (USA); Zamawe 2015 (Malawi).	"If you go to the clinic after giving birth at home, nurses make you pay before they examine your baby" [Sialubanje, Zambia, 2014].	Moderate
			"I took my sick child to the government health centre and nobody was willing to help me… they did not even have medicines. Do you know they said that if we don’t have up to N6000 ($20), they will not attend to the child? [Izugbara 2018, Nigeria]	
	**14. Infant-focused PNC**—In studies from some settings, new mothers were of the view that postnatal care services were largely (or only) directed at the welfare of the infant. Most of these women valued the services on offer and highlighted immunizations and clinical indicators of infant development as particularly useful.	8 studies (1 HIC, 7 LMIC): Alyahaya 2019 (Jordan); Henderson 2016 (USA); Khalaf 2007 (Jordan); Mrisho 2009 (Tanzania); Sacks 2015 (Zambia); Shaban 2018 (Jordan); Sialubanje 2014 (Zambia), Waiswa 2008 (Uganda)	“PNC is just for the child. There is nothing for the mother. All other services that follow soon after birth are for the child.” [Mrisho 2009, Tanzania].	Low
	**15. Gender of healthcare providers**—In studies from some LMIC settings, women felt their needs and sensitivities during the postnatal period were better understood by female health providers and were sometimes ashamed or embarrassed to be seen by a male healthcare provider. In one specific context women felt unsafe if they received a home visit from a male healthcare provider.	5 studies (5 LMIC): Bhattacharyya 2015 (India); Memon 2015 (Pakistan); Newbrander 2014 (Afghanistan); Shaban 2018 (Jordan); Tesfaye 2019 (Ethiopia):	"We are also ashamed of going to male doctors. How can we tell the problems we have to a strange or non-family male?" [Newbrander 2014, Afghanistan]	Low
**EXPERIENCE OF CARE**	**16. Practical support:** Women in included studies from a variety of different HIC contexts and settings expressed the need for practical support to help with the transition to motherhood. During the immediate postpartum period women wanted providers to offer support with infant focused activities like feeding, nappy changing and bathing as well as offering to care for the newborn while mothers recuperated. Although family members often stepped in to offer practical support during the subsequent postnatal stages (at home) some women highlighted a need for ongoing, infant focused support and those without family assistance suggested help with domestic responsibilities (shopping, cooking, cleaning, etc;) might be beneficial.	24 studies (24 HIC): Aune 2012 (Norway); Baker 2005 (UK); Beake 2005 (UK); Cronin 2003 (Ireland); Dahlberg 2016 (Norway); Forster 2008 (Australia), Frei 2011 (Switzerland); Gaboury 2017 (Canada); Henderson 2016 (USA); Hindley 2005 (UK); Humbert 2009 (USA); Kurth 2010 (Switzerland); Leirbakk 2018 (Norway) Lewis 2009 (UK); Munday 2003b (Canada); Noguchi, 2018 (Japan); Persson 2011 (Sweden); Puthussery 2010 (UK); Razurel 2011 (Switzerland); Rouhi, 2019 (Australia); Tully 2017 (USA); Woodward 2016 (Australia); Yeh 2017 (Taiwan); Young 2008 (UK)	"At this [doing the month] stage, physical recovery is essential. I don’t want to keep my baby with me. I believe if I have enough support from midwives, I would become a happy and healthy mother [if I was able to have breaks from caring for my baby]; it also would offer protection against postpartum depression" [Yeh 2017, Taiwan]	High
			"Having no sleep, having no rest and thinking maybe the hospital staff will give the mother a rest by taking the baby away, for a couple of hours would be amazing, but there’s none of that, there was no help, and I actually asked for the help… I think it was the second night I just broke down in tears, because I was so exhausted, in so much agony… And I asked for help and they said look, we don’t do that. I was a bit surprised that, that help wasn’t available. [Puthussery 2010, UK].	
	**17. Psychosocial support**—For many women in the included studies, predominantly in HIC settings, the postnatal period elicited a range of extreme emotions from great joy to exasperation and despair. To cope with these feelings women often received support from family and friends and welcomed frequent reassurance from healthcare providers. Some women expressed a need to discuss their birth with a healthcare provider (ideally someone who was present at the birth) and sometimes they highlighted a need to discuss their anxieties, their fear of responsibility or their perceived insecurities about living up to the ideal of a ’good mother’.	28 studies (25 HIC, 3 LMIC): Aune, 2012 (Norway); Aston, 2018 (Canada); Baker, 2005 (UK); Beake, 2005 (UK); Cronin, 2003 (Ireland); Dahlberg, 2016 (Norway); Forster, 2008 (Australia), Frei, 2011 (Switzerland); Gaboury, 2017 (Canada); George, 2005 (USA); Henderson, 2016 (USA); Hindley, 2005 (UK); Kirca, 2018 (Turkey) Kurth, 2016 (Switzerland); Kurth, 2010 (Switzerland); Leirbakk, 2018 (Norway); McCarter, 2016 (USA); Munday, 2003b (Canada); Newbrander, 2014 (Afghanistan); Noguchi, 2018 (Japan); Persson, 2011 (Sweden); Razurel, 2011 (Switzerland); Ribeiro, 2019 (Brazil); Rouhi, 2019 (Australia); Tully, 2017 (USA); Woodward, 2016 (Australia); Yeh, 2017 (Taiwan); Young, 2008 (UK)	“When I leave, I feel really good, and every time we leave she says “You’re doing a great job, mom. Keep it up” And that just makes me feel so good leaving the office…. that you have a doctor who’s really paying attention to you and your daughter, or your child, and then just confirms for you when you leave, like “Keep it up. You’re doing awesome. She’s healthy, she’s happy". So that is a huge deal" [Aston 2018, Canada].	High
			“I would appreciate if it was part of the routine to have a little talk after the birth…it doesn’t need to be a long talk or so…just as long as you get to meet the midwife who was there”. [Persson 2011, Sweden].	
			"And I think there is a preconceived image of the ideal mother. When I asked friends who gave birth at the same time as me: ‘how is your daughter? Is she crying a lot?’ They all told me no. They said no, she never cries. Recently, I asked them again about it, and they then said that they could not even take a shower! And I said to them: ‘but I thought that she did not cry?’ And even worse, they had not told me the truth, and I found this extremely distressing. I do not know, it is all a facade" [Razurel 2011, Switzerland].	
	**18. Information needs**—For many women in the included studies, largely in HIC contexts, the birth of a newborn triggered a huge array of informational needs. Some of these needs were met by friends, family, peers or the internet but women looked to healthcare providers for specific information about their infant’s feeding, crying or sleeping behaviours as well as clinical information relating to safety, development and overall wellbeing. Women also looked to providers for information about their own needs relating to wound care, the resumption of sex, contraception and their general wellbeing. For some women the amount of information provided was too little or given at the ’wrong’ time (antenatally or immediately after birth) whilst for others the volume of information given by providers was excessive, inappropriate or inconsistent.	31 studies (25 HIC, 6 LMIC): Aune, 2012 (Norway); Aston, 2018 (Canada); Baker, 2005 (UK); Beake, 2005 (UK); Cronin, 2003 (Ireland); Dahlberg, 2016 (Norway); Forster, 2008 (Australia), Frei, 2011 (Switzerland); Gaboury, 2017 (Canada); George, 2005 (USA); Henderson, 2016 (USA); Hindley, 2005 (UK); Khalaf, 2007 (Jordan); Kirca, 2018 (Turkey); Kurth, 2016 (Switzerland); Miteniece, 2018 (Georgia) McCarter, 2016 (USA); Munday, 2003b (Canada); Noguchi, 2018 (Japan); Persson, 2011 (Sweden); Probandari, 2017 (Indonesia); Puthussery, 2010 (UK); Razurel, 2011 (Switzerland); Rodin, 2019 (USA); Rouhi, 2019 (Australia); Shaban, 2018 (Jordan): Tully, 2017 (USA); Woodward, 2016 (Australia); Yeh, 2017 (Taiwan); Young, 2008 (UK); Zamawe, 2015 (Malawi)	"No one gave me information after the delivery. No one gave me information before being discharged" [Kirca 2018, Turkey].	High
			"I had my questions ready, what I wanted to know and I got that information so I was happy. And I had to know what to do if you’ve a problem, that’s the most important thing". [Frei 2011, Switzerland].	
	**19. Acknowledgement of women—**Women in studies from predominantly HIC settings expressed a desire to be ’seen’ by healthcare providers. Most women understood and appreciated the focus of care on their infant’s wellbeing and suppressed or ignored their own need for attention. They were sometimes surprised and relieved when sensitive healthcare staff enquired about their wellbeing but, more often than not, felt disappointed, unsupported and uncared for when their need for attention or simply to be acknowledged was ignored or overlooked.	20 studies (18 HIC, 2 LMIC): Aune 2012 (Norway); Aston, 2018 (Canada); Baker 2005 (UK); Beake, 2005 (UK); Cronin 2003 (Ireland); Dahlberg 2016 (Norway); Forster 2008 (Australia), Frei 2011 (Switzerland); Gaboury 2017 (Canada); Hindley 2005 (UK); Khalaf 2007 (Jordan); Kurth, 2016 (Switzerland); McCarter 2016; Mrisho 2009 (Tanzania); (USA); Munday 2003a (Canada); Persson 2011 (Sweden); Puthussery 2010 (UK); Rodin 2019 (USA); Tully 2017 (USA); Woodward 2016 (Australia)	"That someone came to your home to ask you how YOU were doing was something I appreciated. Everyone tended to ask about the baby, but suddenly there was someone who wanted to know how WE were doing after the birth". [Dahlberg 2016, Norway].	High
			"It’s kind of disheartening when they come in and ask for (baby) first, because I’m hurting too” [Gaboury, 2017 Canada].	
	**20. Continuity of carer**—Women in studies from a variety of different HIC settings and contexts highlighted the importance of forming a trusting relationship with a healthcare provider. For some women this entailed seeing the same provider for all aspects of maternity care (from antenatal to postnatal), for others it was important to receive postnatal care from a provider who was present at the birth, and, for a few, simply seeing the same person at each postnatal contact was a key component of quality care. For women who chose to have homebirths the prospect of seeing the same midwife throughout their maternity journey was an important factor in their decision-making.	12 studies (11 HIC, 1 LMIC): Alyahya, 2019 (Jordan); Aune 2012 (Norway); Dahlberg 2016 (Norway); Fre, 2011 (Switzerland); Hindley 2005 (UK); Munday 2003b (Canada); Noguchi 2018 (Japan); Persson 2011 (Sweden); Puthussery 2010 (UK); Rodin 2019 (USA); Tully 2017 (USA); Woodward 2016 (Australia)	"If there had been another midwife who came at the home visit, I would not have had the same experience. The main thing was that it was exactly her, so that we could continue where we left off. She knew how I felt during pregnancy. I saw that she was excited about how I was doing with my baby, so it was more than just a medical check-up, and that felt very good" [Dahlberg 2016, Norway].	Moderate
			‘‘I saw maybe two or three different people over the four or five times that I went… it just wasn’t the same person and I wanted the same person all the time.” [Woodward 2016, Australia]	
	**21. Disrespect and abuse—**Women from studies in a variety of different countries and contexts reported rude and abusive behaviour by healthcare providers. As well as a general lack of respect women reported acts of discrimination and humiliation and verbal and physical abuse during their PNC encounters. In some contexts, women were scolded or punished by healthcare providers for giving birth at home.	8 studies (3 HIC, 5 LMIC): Baker, 2005 (UK); Bhattacharyya, 2015 (India); Hindley, 2005 (UK); Humbert, 2009 (USA); Izugbara, 2018 (Nigeria); Kanengoni, 2019 (Zimbabwe); Mrisho, 2009 (Tanzania); Sialubanje, 2014 (Zambia)	"I actually heard one (midwife) call one mother a crybaby because she’d had a caesarean and she had stitches. She was puffy and bruised and sick and because she couldn’t immediately jump up and tend to the baby whenever she cried they, they called her crybaby because she would buzz for help because she couldn’t physically get to the child and do what was needed". [Baker 2005, UK]. "	Moderate

### Themes identified from included studies

#### Access and availability

Whilst proximity to a health facility appeared to encourage engagement with maternity providers, our evidence suggests that, for some women living in remote or rural areas, a lack of transport or the poor quality of transport networks limited attendance at postnatal clinics. This was compounded in situations where women did not have the personal resources to pay for relatively expensive journeys to health facilities and/or could not afford to take time away from their work or family. Even in high income settings where access to postnatal services is ostensibly free at point of care, the additional costs associated with attendance including insurance levies, childcare costs, and transport costs limited engagement for women living in poverty.

For accessing postnatal care post-discharge from a health facility after birth, women wanted a wide range of possible options and flexible schedules for reaching healthcare workers. Women generally valued the ability to contact providers at convenient times even more so than having a large number of contacts. Women wanted to be able to get support during moments of high stress, or on their schedules, rather than on a pre-defined health systems schedule, and many referenced the value of their time. Women expressed frustration about not being able to reach healthcare workers when needed. Service providers that were able to offer more flexible opportunities for engagement like drop-in clinics, telephone contacts, out of hours services and, in particular, home visits, were viewed more positively.

#### Physical and human resources

For women in a variety of different settings, the ability to engage with formal postnatal services was influenced by resource and infrastructure constraints, especially in settings where community-based services were limited or non-existent. The evidence also suggests that the poor availability of resources in some health facilities may act as a deterrent to women who might otherwise benefit from postnatal care. A lack of basic medicine and equipment and inadequate or inconsistent water or electricity supplies limited attendance in some low-income settings. Whilst the availability of essential equipment and utilities was not reported to be an issue in most high-income countries, women were sometimes aware of staff shortages on postnatal wards and this affected their experience of care. Women’s perception that some health facilities were understaffed, especially from studies in LMICs, was also reflected in the length of time they had to wait to be seen by a healthcare provider. In some instances, this was compounded by cursory and impersonal exchanges with care providers, leaving women feeling frustrated, annoyed and undervalued.

#### External influences

Women identified several external influences as having a bearing on their engagement with postnatal services. These ranged from environmental influences such as the physical condition of the health facility itself to the availability and affordability of private providers to a willingness (or otherwise) to engage with traditional postnatal practices, either in accordance with or against the advice of family and community members.

For women in a variety of different settings and contexts, the condition of postnatal wards and health facilities was important. Women used words such as ‘clean’ and ‘modern’ to frame positive perceptions or ‘dirty’ and ‘unhygienic’ to highlight negative experiences. These negative accounts were more commonly associated with facilities in low-income settings but even in high income countries women used words like ‘dilapidated’ and ‘unwelcoming’ to describe postnatal wards. In addition to the condition of the buildings, women also commented on the lack of physical space in some facilities and how this impacted on their sense of personal space and perception of privacy. Some women felt the opportunity to engage in confidential conversations with family members or healthcare providers was compromised whilst others felt the shared facilities and tight surroundings in some postnatal wards generated a noisy and disruptive atmosphere. For mothers who already felt exhausted and fatigued from childbirth, the impact of this environment coupled with their inability to control system-oriented, organizational routines, led to feelings of frustration and exasperation.

By contrast, for women who gave birth at home, the nurturing nature of familiar surroundings as well as their ability to establish personal routines and control access to their home created a more relaxing environment. In settings where private facilities were available, they were generally considered to be of better quality and were utilised by some women with the financial means to do so. However, in some contexts, the integration between private and public providers was inadequate and impacted on women’s engagement with postnatal services once they were discharged from the health facility.

Women’s capacity to engage with postnatal services was influenced by other family members and individuals in their social circles. In some contexts, women’s autonomy was inhibited by patriarchal social structures and decisions relating to engagement with maternity services, including postnatal care, were largely deferred to husbands. Sometimes, these kinds of decisions were agreed jointly between the woman’s husband and her mother-in-law and sometimes the decision was solely the responsibility of the mother-in-law.

Women expressed that elderly relatives and the broader beliefs and expectations of local communities influenced their observance of traditional postnatal practices rather than ‘westernised’ approaches to postnatal care, which some may have preferred. In some rural communities, especially in Africa, the reliance on TBAs to administer specific herbs and medicines in the postnatal period was integral to a communal belief system, whilst in other settings it was simply more convenient or financially viable. For other women, especially in Asia, the cultural practice of ‘doing the month’ involved extended periods of isolation and seclusion and limited interaction with formal postnatal services. Our findings also indicate that, in these contexts, some women (and their families) found it difficult to steer a course between the increasing influence of “Western” approaches to postnatal care and adhering to the traditional practices advocated by previous generations.

#### Social norms

Women highlighted a variety of behaviours and understandings about the health system that affected their willingness to engage with postnatal care providers. For some women, especially from studies in LMICs, these understandings were based on a perception that attendance at health facilities offering postnatal care was only necessary if they felt unwell or if there was a problem with their infant. In many cases, this notion was reinforced by healthcare providers who did not encourage attendance or devalued the services they offered. When health workers devalued PNC, families also tended to devalue PNC and not see the need to seek care.

Some women also believed that postnatal services were solely focused on infant wellbeing and development and, although they valued the services on offer for newborns (clinical assessments and immunizations), they were not aware of, or did not acknowledge, any sources of care and support for themselves.

For some women, a reluctance to engage with postnatal services was rooted in a lack of trust in the system. In certain contexts, this was based on a perception that some providers were corrupt and expected informal payments, gifts, or bribes in return for care. In other settings, women’s trust in the system was undermined by perceived inadequacies in the clinical or personal skills of the healthcare providers. More infrequently, women complained that confidential information shared with health providers might be compromised or abused and, in more extreme cases, women believed that disclosure of mental health issues (like postnatal depression) might lead to their infant being taken away from them. In a few specific contexts, women expressed a preference to be seen by female health providers and highlighted safety concerns when postnatal visits at home were conducted by male health workers.

#### Experience of care

Based on their experiences of postnatal care, women identified a range of issues that were of particular importance during their postnatal journey, including the need for information and support and the desire to be treated with care and respect by familiar and trusted healthcare providers.

Women from a variety of different settings and contexts highlighted the need for information during all phases of postnatal care. Although some of these informational needs were met by friends, peers, family members and online sources, women looked to healthcare providers for information about infant nutrition and development as well as tips and advice on infant crying cues, sleeping patterns, breastfeeding, and safety concerns. Although women tended to prioritise the needs of their newborns over their own, they also sought personal information for example on wound care, contraception, and when to resume sexual activity. The timing and delivery of information was also discussed by many women indicating that information should be supplied both antenatally and postnatally and given in a clear and consistent format. For some women, intense emotions of joy and elation coupled with feelings of extreme fatigue affected their ability to absorb information in the immediate postnatal period, whilst for others, the sheer volume of information was difficult to process.

In addition to a need for information, women also identified needs for both practical support and, especially in high-income countries, for psychosocial support. In a practical sense, women appreciated the support they received from family members but also valued support from healthcare providers, particularly in the immediate postpartum period, prior to hospital discharge, when they were trying to bond with their newborn and/or establish breastfeeding. Help with specific newborn-oriented tasks like nappy changing and bathing as well as tending to the newborn whilst the mothers recuperated, showered, or carried out chores, were highlighted and, in some instances, women felt disappointed when these needs were not recognised.

In many settings, women also highlighted the need for ongoing practical support once they returned home and, although this was often facilitated by family members, women also appreciated assistance from healthcare providers during the transition to motherhood. Usually this was a continuation of the advice received in hospital relating to infant feeding and development but, in uncommon circumstances, women received visits from associated agency workers to helped with domestic activities (shopping, cleaning, cooking) and these services were highly valued.

Many women experienced intense emotional peaks and troughs during the postpartum period ranging from elation to despair to overwhelming exhaustion. Women, particularly first-time mothers, discussed their fears, anxieties, and insecurities about becoming a mother and, for some, the pressure and responsibility of living up to some idealised version of a mother. Women wanted support from healthcare providers to help them to process and manage these difficult emotions and often expressed this in terms of a need for reassurance. In some contexts, particularly in high-income settings, where much of the published evidence comes from, women wanted to discuss the birth experience with a midwife who was present or have access to healthcare providers support if they felt their birth was challenging or traumatic.

In a broader sense, many women felt that their own care needs were overlooked or undervalued during the postpartum period. Whilst new mothers completely accepted and understood that the focus of postnatal care was on their infant, they nevertheless felt disappointed when unvoiced pleas for attention or recognition were ignored by healthcare providers.

Our findings also indicate that women placed great importance on their ability to build a relationship with care providers and this was particularly apparent in high-income settings. For some women this involved seeing the same healthcare provider at each postnatal contact, for others it meant being able to see the same midwife during the postnatal period as they saw antenatally, and for women who gave birth at home, the prospect of having the same midwife throughout their maternity journey played a significant role in their decision to opt for a homebirth. Where women were able to build these relationships, they were more likely to report ‘a sense of companionship’, ‘trust’ and ‘authenticity’, but in settings where continuity of healthcare models were not in place, women reported feeling ‘dissatisfied’, ‘like a number’ or even, ‘like an animal’.

For women in several contexts, interactions with healthcare providers sometimes became disrespectful and abusive. In high income settings, women indicated that healthcare providers could be rude or undermining and occasionally discriminatory during postnatal encounters, whilst in lower-income contexts women reported acts of rudeness, humiliation and, in rare cases, punishment by health providers.

## Discussion

Factors that influence women’s utilization of postnatal care are interlinked, and include access, quality, and social norms. Five review findings were identified: access and availability; physical and human resources; external influences; social norms; and experience of care. Many women recognised the specific challenges of the postnatal period and emphasised the need for emotional and psychosocial support in this time, in addition to clinical care.

Staffing and resources were important to women, although in low-resource settings, more emphasis was placed on poor physical infrastructure. In low- and middle-income countries, women further expressed that healthcare providers themselves often devalued postnatal care, contributing to their lack of utilization and sense of unpreparedness. Many studies from high-income countries highlighted women’s desire for more psychosocial and emotional support; yet, women in low income settings may not have been asked as directly about this challenge. Women also may not believe this is a role of the health system, or may not feel comfortable stating this as a vulnerability. These findings point to the need to strengthen comprehensive health care services, which can more fully address the holistic and ongoing needs of women and their families.

Many of the findings related to experience of care derived from high-income countries. Because of the number of included studies related to this topic were biased toward high income countries, this review finding should not be interpreted necessarily as women in low- and middle-income countries having positive experiences of care; evidence indicates that disrespectful practices are common globally [93]. This area is understudied in low- and middle-income countries and therefore it is difficult to draw robust conclusions. However, it is likely that women in settings with insufficient resources will more often refer to unhygienic conditions or lack of equipment as a more immediate priority than their experiences, and/or that they perceive less ability to change the situation than women in settings with more resources. A recent qualitative evidence review of studies in sub-Saharan Africa affirmed that aspects of respectful and disrespectful maternity care and women’s previous experiences of health care influenced their “decisions to access postnatal care services” [94]. The fact that many of the studies related to experience of care are from high-income settings may reflect the study authors’ biases and points to the need to study women and families’ experiences more holistically in low- and middle-income settings.

When situating this review within the context of other research [29], many similarities emerge in review findings across various phases of maternity care. From antenatal and intrapartum through postnatal care, women emphasised the need for information, continuity of care, adequate resources, and comprehensive and holistic support. Access and cost continue to be issues for many women, especially in low- and middle-income countries and in rural areas, but compared with intrapartum care, the incentive to overcome these challenges is further diminished with the devaluing of postnatal care and perception of low need for healthy women and their healthy infants. In the postnatal period, women’s access needs include when and how they can contact healthcare providers and for what purposes. Women greatly value continuity of care and flexible schedules for obtaining information and assistance. Infrastructure and health system resources play into both decisions about if and when to seek care, as well as the experience of care itself. This pattern and commonality across maternity care periods reflects the fact women may seek care from the same places and thus experience some of the same facilitators and challenges, but also emphasises women’s perception that maternity and the postnatal period are a continuum. The factors influencing postnatal care utilization may be different than other maternal and child health services for a number of reasons: postnatal may not be seen as important (especially if the woman and newborn are apparently healthy); during the postnatal care period, maternal and newborn needs may arise at the same time, adding to complexity of recognition and care seeking; and health care visits may take place in the home, unlike visits which must take place in a health facility. However, many of the same factors may be at play, including the recognition of need, the perception of quality, and the physical barriers such as cost and distance.

The review findings on postnatal care utilization largely conform with previous studies around what women want during this time period, as well as challenges related to access, health system quality, and experience of care. Our review builds on previous work in postnatal care utilization by explicitly including both women and newborns. The strengths of this review include a rigorous methodology, comprehensive search, very large database, wide search terms and concepts, and a diverse study team. Our review encompassed a geographically and linguistically diverse search, with a balance of papers from high, middle, and low-income countries, although the number of available studies from certain regions (e.g. Latin America and the Eastern Mediterranean) were limited. Despite the design of the search to be global, including a lack of language restrictions, we identified few papers from Australasia, Middle East, and South America.

Some potential limitations of the study include the limitations of the included papers themselves, especially the different prioritised topics studied in different regions of the world. Although the objectives of the included papers represented a range of topics, it is possible that certain areas, as well as certain topics in each region, are understudied. While we acknowledge that there may be context specific issues, we are bound by the content of the included studies and recognise that different questions may have been posed to participants in different contexts, depending on the nature of the research inquiry and the pre-existing beliefs of the research team members of those particular studies. Further, the World Bank Country Classifications are broad and group countries with very different profiles together. Country-specific terminology (such as the specific words used in a particular setting around health insurance or a certain cadre of support worker) may not have been captured.

As with other systematic reviews, there is a trade-off between speed and comprehensiveness and, while our use of sampling could limit our interpretation, our iterative process until reaching saturation increases confidence in our findings [95, 96]. New studies have been published since the end of the search that were not included, however, the comprehensiveness and rigor of our search and analysis provides confidence in the findings.

Many papers identified in our search included the term “postnatal care” but in fact referred only to intrapartum care. It was difficult to disentangle experiences of postnatal care by time period as this was rarely disaggregated in studies. The differentiation was included in our extraction form, but some papers reported on when data were collected and others on the period the respondents were referring to with the latter often encompassing multiple time points post birth. More research is needed in distinguishing the needs during the immediate (e.g. pre-discharge from a health facility) and later postnatal periods.

The findings from this study have implications at the individual, family, health system, and policy levels, and interventions may be needed to address factors at each. Individual empowerment of women may be insufficient if her partner, family, or community have significant influence in healthcare decisions. The desire of women to have increased emotional and psychosocial support may or may not be best served by existing cadres of medical providers. Future research should explore who the optimal providers might be and what the scope (and burden) might be for each type of provider, including traditional birth attendants [97] and non-medical carers. The intervening time from the end of our search to completion of analysis included the emergence of a global pandemic, which has already had significant impact on postnatal experiences and care utilization [98, 99]. Further areas of research include the impact of the pandemic on care utilization, increased anxiety and psychosocial support needs [100], and the role of digital and virtual care technologies [101].

There are clear steps which can be taken to improve the quality, experience, and uptake of care for women and newborns in the postnatal period. The value of PNC should be promoted as part of quality improvement, health worker training, and community mobilization. As much as possible, care should be provided in a continuous and coordinated manner, between health facilities, clinics, medical offices, communities, and households. At each level there must be sufficient staffing, resources, and infrastructure to provide high quality of care. Efforts should be taken to eliminate barriers to cost and transport, including illegal or unethical barriers such as bribes and other out-of-pocket or unanticipated costs for care, and all types of abuse and denial of care.

## Conclusions

Postnatal care must be positioned as a high priority for both the woman and the newborn, much like antenatal and intrapartum care, and not seen as an optional service, or one only accessed in cases of emergencies. As a pre-requisite for increased utilization of postnatal care, quality must be improved [102]. The benefit of postnatal care for the mother and entire family may increase utilization, especially if services are available to improve emotional and psychosocial support. The implementation of standards for quality of care and respectful care must move beyond childbirth to ensure a positive experience of postnatal care for all women and their newborns.

## Supporting information

S1 Appendix Full search strategy. (DOCX)

S2 Appendix *A priori* framework. (DOCX)

S1 Checklist PRISMA 2020 checklist. (DOCX)

## Abbreviations

LMIC: Low- or Middle-Income Countries
MeSH: Medical Subject Headings
PNC: Postnatal Care
PRISMA: Preferred Reporting Items for Systematic Review and Meta-Analyses
WHO: World Health Organisation
