# Supplementary material for: Factors that influence uptake of routine postnatal care: Findings on women’s perspectives from a qualitative evidence synthesis
Source: PLoS One. 2022 Aug 12;17(8):e0270264. doi: 10.1371/journal.pone.0270264 (PMC9374256; doi:10.1371/journal.pone.0270264)
Supplement: S1 Appendix — (DOCX) [file pone.0270264.s001.docx]

**Appendices**

1. Full search strategies

|  | **PUBMED** | **Results** |
| --- | --- | --- |
|  | QUERY |  |
| 1 | ("Communication Barriers"[Majr:NoExp] OR "Healthcare Disparities"[Majr:NoExp] OR "Social Determinants of Health"[Majr:NoExp] OR "Socioeconomic Factors"[Majr:NoExp] OR "Patient Acceptance of Health Care"[Majr:NoExp] OR aided[ot] OR aided[ti] OR aiding[ot] OR aiding[ti] OR barricade*[ot] OR barricade*[ti] OR barrier[ot] OR barrier[tiab] OR barriers[ot] OR barriers[tiab] OR block*[ot] OR block*[ti] OR challeng*[ot] OR challeng*[ti] OR constrain*[ot] OR constrain*[ti] OR curtail*[ot] OR curtail*[ti] OR determinant*[ot] OR determinant*[tiab] OR difficult*[ot] OR difficult*[tiab] OR discourag*[ot] OR discourag*[tiab] OR disincentive*[ot] OR disincentive*[ti] OR disincline*[ti] OR encourag*[ot] OR encourag*[ti] OR encumber*[ti] OR encumbrance*[ot] OR encumbrance*[ti] OR enhanc*[ot] OR enhanc*[ti] OR facilitat*[ot] OR facilitat*[tiab] OR gaps[ot] OR gaps[ti] OR glitch*[ot] OR glitch*[ti] OR hamper*[tiab] OR hamper*[ti] OR hangup[ti] OR hang-up[ti] OR help*[ot] OR help*[ti] OR hinder*[ot] OR hinder*[ti] OR hindrance*[ot] OR hindrance*[ti] OR hold-up*[ot] OR hold-up*[ti] OR impair*[ot] OR impair*[ti] OR impede*[ot] OR impede*[ti] OR impediment*[ot] OR impediment*[ti] OR impeding[ot] OR impeding[tiab] OR influence[ot] OR influence[tiab] OR influenced[ot] OR influenced[tiab] OR influencing[ot] OR influencing[tiab] OR interdict*[ot] OR interdict*[ti] OR interfer*[ot] OR motivat*[ti] OR interfer*[ti] OR limit[ot] OR limit[ti] OR access*[ot] OR access*[ti] OR motivat*[ot] OR neglect*[ti] OR neglect*[ti] OR non-attendance[ot] OR non-attendance[ti] OR objected[ot] OR objected[ti] OR objecting[ti] OR obstacle*[ot] OR obstruct*[ot] OR obstruct*[ti] OR obstacle*[ti] OR attendance[ot] OR attendance[ti] OR problem*[ot] OR problem*[ti] OR prohibit*[ot] OR prohibit*[ti] OR promot*[ot] OR promot*[ti] OR reduce[ot] OR reduce[ti] OR reducing[ot] OR reducing[ti] OR refus*[ot] OR refus*[ti] OR reject*[ot] OR reject*[ti] OR reject*[ti] OR reject[ot] OR restrain*[ot] OR restrain*[ti] OR restrain*[ti] OR restrict*[ot] OR restrict*[tiab] OR roadblock*[ot] OR roadblock*[ti] OR stop*[ot] OR stop*[ti] OR unwilling[ot] OR unwilling[ti] OR willing[ot] OR willing[ti] OR uptake*[tiab] OR uptake*[ot] OR limits[ot] OR limits[tiab] OR limited[ot] OR limited[tiab] OR limiting[ot] OR limiting[tiab] OR support*[ti] OR experience[ti] OR support*[ot] OR experience[ot] OR "Health Services Accessibility"[Majr:NoExp]) | 5838377 |
| 2 | ("Postnatal Care"[Majr:NoExp] OR "Postpartum Period"[Majr:NoExp] OR post-partum[tiab] OR post-partum[ot] OR postpartum[ot] OR postpartum[tiab] OR post-natal[tiab] OR post-natal[ot] OR postnatal[ot] OR postnatal[tiab] OR puerperium[tiab] OR puerperium[ot] OR puerperal[ot] OR puerperal[tiab] OR postparturient[tiab] OR post-parturient[tiab] OR postparturient[ot] OR postparturition[tiab] OR postparturition[ot] OR post-parturition[tiab] OR afterbirth[tiab] OR afterbirth[ot] OR maternity care[tiab] OR maternity care[ot] OR neonatal care[tiab] OR neonatal care[ot] OR neonatal health[tiab] OR neonatal health[ot] OR maternity health[tiab] OR maternity health[ot] OR "maternal and child health"[tiab] OR "maternal and child health"[ot] OR "Infant, Newborn"[Majr:NoExp] OR "Maternal Health Services"[Majr:NoExp] OR "Maternal Mortality"[Majr:NoExp] OR "Maternal-Child Health Centers"[Majr:NoExp] OR "Maternal-Child Health Services"[Majr:NoExp] OR "Maternal Welfare"[Majr:NoExp] OR maternal health service*[tiab]) | 221118 |
| 3 | (“Case Reports”[Mesh] OR “Organizational Case Studies”[Mesh] OR action research[tiab] OR “Community-Based Participatory Research”[Mesh] OR participatory research[tiab] OR case stud*[tiab] OR ethno*[tiab] OR grounded theory[tiab] OR phenomeno*[tiab] OR “Narration”[tiab] OR narrative*[tiab] OR biograph*[tiab] OR biograph*[tiab] OR “Autobiography”[Mesh] OR autobiograph*[tiab] OR documentar*[tiab] OR qualitative synthes*[tiab] OR active feedback[tiab] OR conversation*[tiab] OR discourse*[tiab] OR thematic[tiab] OR key informant*[tiab] OR "Focus Groups"[Mesh] OR Focus Group*[tiab] OR case report*[tiab] OR “Interview”[Mesh] OR interview*[tiab] OR “Observation”[Mesh] OR observer*[tiab] OR visual data[tiab] OR audio record*[tiab] OR “Anthropology, Cultural”[Mesh] OR “Comparative Study”[Mesh] OR comparative stud*[tiab] OR comparative stud*[tiab] OR “Evaluation Studies”[Mesh] OR evaluation stud*[tiab] OR mixed method*[tiab] OR multimethod*[tiab] OR multiple method*[tiab]) | 1467459 |
| 4 | ("Attitude to Health"[Mesh] OR "Attitude of Health Personnel"[Mesh] OR "Ceremonial Behavior"[Mesh] OR "Choice Behavior"[Mesh] OR "Community Health Services"[Mesh] OR "Community Health Workers"[Mesh] OR "Continuity of Patient Care"[Mesh] OR "Culture"[Mesh] OR "Cultural Characteristics"[Mesh] OR "Directive Counseling"[Mesh] OR "Health Behavior"[Mesh] OR "Health Knowledge, Attitudes, Practice"[Mesh] OR "Health Services Needs and Demand"[Mesh] OR attitude*[tiab] OR attitude*[ot] OR behavior*[ti] OR behavior*[ot] OR Cultur*[ti] OR Cultur*[ot] OR knowledge[tiab] OR knowledge[ot]) | 2295302 |
| 5 | **1 AND 2 AND 3 AND 4** | **4567** |
|  | **MEDLINE** | **Results** |
| 1 | *Communication Barriers/ or  *Healthcare Disparities/ or  *Social Determinants of Health/ or  *Socioeconomic Factors/ or  *Patient Acceptance of Health Care/ or  *Health Services Accessibility/ or  (aided or aiding or barricade* or block* or challeng* or constrain* or curtail* or disincentive* or encourag* or encumbrance* or enhanc* or gaps or glitch* or hang?up.ti,bt.help* or hinder* or hindrance* or hold-up* or impair* or impede* or impediment* or interdict* or motivat* or interfer* or limit or access* or non-attendance or objected or obstruct* or obstacle* or attendance or problem* or prohibit* or promot* or reduce or reducing or refus* or reject* or reject* or restrain* or restrain* or roadblock* or stop* or unwilling or willing or support* or experience).ti,bt,kw  or  (barrier or barriers or determinant* or difficult* or discourag* or facilitat* or impeding or influence or influenced or influencing or uptake* or limits or limited or limiting or restrict*).ti,ab,cl,oa,kw,kf  or  hamper*.ti,ab,cl,oa,kw,kf,bt  or  (disincline* or encumber* or neglect* or objecting).ti,bt | **5791904** |
| 2 | *Postnatal Care/ or  *Postpartum Period/ or  *Infant, Newborn/ or  *Maternal Health Services/ or  *Maternal Mortality/ or  *Maternal-Child Health Centers/ or  *Maternal-Child Health Services/ or  *Maternal Welfare/ or  (post?partum or post?natal or puerperium or puerperal or post?parturient or post?parturition or afterbirth or maternity care or neonatal care or neonatal health or maternity health or "maternal and child health" or maternal health service*).ti,ab,cl,oa,kw,kf. | 221976 |
| 3 | (action research or participatory research or case stud* or ethno* or grounded theory or phenomeno* or Narration or narrative* or biograph* or biograph* or autobiograph* or documentar* or qualitative synthes* or active feedback or conversation* or discourse* or thematic or key informant* or Focus Group* or case report* or interview* or observer* or visual data or audio record* or comparative stud* or comparative stud* or evaluation stud* or mixed method* or multimethod* or multiple method*).ti,ab,cl,oa,kw,kf.  or  exp "Case Reports"/ or  exp "Organizational Case Studies"/ or  exp "Community-Based Participatory Research"/ or  exp Focus Groups/ or  exp "Interview"/ or  exp "Observation"/ or  exp "Anthropology, Cultural"/ or  exp "Comparative Study"/ or  exp "Evaluation Studies"/ or  exp "Autobiography"/ | 5092830 |
| 4 | (attitude* or knowledge).ti,ab,cl,oa,kw,kf.  or  (behavior* or culture*).ti,bt,kw  or  exp Attitude to Health/ or  exp Attitude of Health Personnel/ or  exp Ceremonial Behavior/ or  exp Choice Behavior/ or  exp Community Health Services/ or  exp Community Health Workers/ or  exp Continuity of Patient Care/ or  exp Culture/ or  exp Cultural Characteristics/ or  exp Directive Counseling/ or  exp Health Behavior/ or  exp Health Knowledge, Attitudes, Practice/ or  exp "Health Services Needs and Demand"/ | 2256071 |
| 5 | **1 and 2 and 3 and 4** | **5418** |
|  | **EMBASE** |  |
| 1 | (aided or aiding or barricade* or block* or challeng* or constrain* or curtail* or disincentive* or encourag* or encumbrance* or enhanc* or gaps or glitch* or help* or hinder* or hindrance* or hold-up* or impair* or impede* or impediment* or interdict* or motivat* or interfer* or limit or access* or non-attendance or objected or obstruct* or obstacle* or attendance or problem* or prohibit* or promot* or reduce or reducing or refus* or reject* or restrain* or roadblock* or stop* or unwilling or willing or support* or experience).ti,bt,kw  or  (impeding or influence or influenced or influencing or restrict* or uptake* or limits or limited or limiting orbarrier or barriers or determinant* or difficult* or discourag* or facilitat*).ti,ab,kw.  or  (disincline* or encumber* or hang?up or neglect*).ti,bt  or  hamper*.ti,ab,kw,bt  or  *communication barrier/ or  *health care disparity/ or  *"social determinants of health"/ or  *socioeconomics/ or  *patient attitude/ or  *patient attitude/ | **7226463** |
| 2 | (post?partum or post?natal or puerperium or puerperal or post?parturient or post?parturition or afterbirth or maternity care or neonatal care or neonatal health or maternity health or "maternal and child health" or maternal health service*).ti,ab,kw.  or  *postnatal care/ or  *puerperium/ or  *newborn/ or  *maternal health service/ or  *maternal mortality/ or  *maternal child health care/ or  *maternal welfare/ | 271031 |
| 3 | (action research or participatory research or case stud* or ethno* or grounded theory or phenomeno* or narration or narrative* or biograph* or biograph* or autobiograph* or documentar* or qualitative synthes* or active feedback or conversation* or discourse* or thematic or key informant* or Focus Group* or case report* or interview* or observer* or visual data or audio record* or comparative stud* or comparative stud* or evaluation stud* or mixed method* or multimethod* or multiple method*).ti,ab,kw.  or  exp case report/ or  exp health services research/ or  exp participatory research/ or  exp interview/ or exp observation/ or  exp cultural anthropology/ or  exp comparative study/ or  exp comparative study/ | **4931420** |
| 4 | (attitude* or knowledge).ti,ab,kw.  or  (behavior* or cultur*).ti,bt,kw  or  exp attitude to health/ or  exp health personnel attitude/ or  exp symbolism/ or  exp decision making/ or  exp community care/ or  exp health auxiliary/ or  exp patient care/ or  exp cultural factor/ or  exp directive counseling/ or  exp health behavior/ or  exp health service/ | 6719686 |
| 5 | **1 and 2 and 3 and 4** | 7187 |
|  | **EBM-REVIEWS** |  |
| 1 | (aided or aiding or barricade* or block* or challeng* or constrain* or curtail* or disincentive* or encourag* or encumbrance* or enhanc* or gaps or glitch* or help* or hinder* or hindrance* or hold-up* or impair* or impede* or impediment* or interdict* or motivat* or interfer* or limit or access* or non-attendance or objected or obstruct* or obstacle* or attendance or problem* or prohibit* or promot* or reduce or reducing or refus* or reject* or restrain* or roadblock* or stop* or unwilling or willing or support* or experience).ti,bt,kw  or  (impeding or influence or influenced or influencing or restrict* or uptake* or limits or limited or limiting orbarrier or barriers or determinant* or difficult* or discourag* or facilitat*).ti,ab,kw.  or  (disincline* or encumber* or hang?up or neglect*).ti,bt  or  hamper*.ti,ab,kw,bt | 405369 |
| 2 | (post?partum or post?natal or puerperium or puerperal or post?parturient or post?parturition or afterbirth or maternity care or neonatal care or neonatal health or maternity health or "maternal and child health" or maternal health service*).ti,ab,kw. | 14153 |
| 3 | (action research or participatory research or case stud* or ethno* or grounded theory or phenomeno* or narration or narrative* or biograph* or biograph* or autobiograph* or documentar* or qualitative synthes* or active feedback or conversation* or discourse* or thematic or key informant* or Focus Group* or case report* or interview* or observer* or visual data or audio record* or comparative stud* or comparative stud* or evaluation stud* or mixed method* or multimethod* or multiple method*).ti,ab,kw. | 107220 |
| 4 | (attitude* or knowledge).ti,ab,kw.  or  (behavior* or cultur*).ti,bt,kw | 84495 |
| 5 | 1 and 2 and 3 and 4 | 212 |
|  | **CINAHL** |  |
| 1 | aided OR aiding OR barricade* OR barrier OR barriers OR block* OR challeng* OR constrain* OR curtail* OR determinant* OR difficult* OR discourag* OR disincentive* OR disincline* OR encourag* OR encumbrance OR enhance* OR facilitat* OR gaps OR glitch* OR hamper* OR hangup OR hang-up OR help* OR hinder* OR hindrance* OR hold-up* OR impair* OR impede* OR impediment* OR impeding OR influence OR influenced OR influencing OR interdict* OR interfer* OR motivat* OR limit OR access* OR motivat* OR neglect* OR non-attendance OR objected OR obstacle* OR obstruct* OR attendance OR problem* OR prohibit* OR promot* OR reduce OR reducing OR refus* OR reject* OR reject* OR restrain* OR restrain* OR restrict* OR roadblock* OR stop* OR unwilling OR willing OR uptake* limits OR limited OR limiting OR support* OR experience OR (MM "Health Services Accessibility") OR (MM "Socioeconomic Factors") OR (MM "Social Determinants of Health") OR (MM "Healthcare Disparities") OR (MM "Communication Barriers") | 2,284,581 |
| 2 | post-partum OR postpartum OR post-natal OR postnatal OR puerperium OR puerperal OR postparturient OR post-parturient OR postparturient OR postparturition OR post-parturition OR afterbirth OR maternity care OR neonatal care OR neonatal health OR maternity health OR "maternal and child health" OR maternal health service* OR MM "Postnatal Care" OR MM "Postnatal Period" OR MM "Infant, Newborn" OR MM "Maternal Health Services" OR MM "Maternal Mortality" OR MM "Maternal-Child Health" OR MM "Maternal Welfare" OR MM "Maternal-Child Welfare" | 62,573 |
| 3 | action research OR participatory research OR case stud* OR ethno* OR grounded theory OR phenomeno* OR “Narration” OR narrative* OR biograph* OR biograph* ORautobiograph* OR documentar* OR qualitative synthes* OR active feedback OR conversation* OR discourse* OR thematic OR key informant* OR Focus Group* OR case report* OR interview* OR observer* OR visual data OR audio record* OR comparative stud* OR comparative stud* OR evaluation stud* OR mixed method* OR multimethod* OR multiple method* OR MH "Case Studies" OR MH "Focus Groups" OR MH "Observational Methods+" OR MH "Anthropology, Cultural" OR MH "Comparative Studies+" OR MH "Evaluation Research+" OR MH "Autobiographies" OR MH "Interviews+" | 823,161 |
| 4 | attitude* OR behavior* OR Cultur* OR knowledge OR MH "Attitude to Health+" OR MH "Attitude of Health Personnel+" OR MH "Community Health Services+" OR MH "Community Health Workers" OR MH "Continuity of Patient Care+" OR MH "Culture+" OR MH "Health Behavior+" OR MH "Health Services Needs and Demand+" | 1,332,743 |
| 5 | **S1 AND S2 AND S3 AND S4** | 7111 |
