# Supplementary material for: Factors that influence uptake of routine postnatal care: Findings on women’s perspectives from a qualitative evidence synthesis
Source: PLoS One. 2022 Aug 12;17(8):e0270264. doi: 10.1371/journal.pone.0270264 (PMC9374256; doi:10.1371/journal.pone.0270264)
Supplement: S2 Appendix — (DOCX) [file pone.0270264.s002.docx]

**Appendices**

1. *A priori* framework

| **THEMES** | **POTENTIAL EXAMPLES** |
| --- | --- |
| **RESOURCES AND ACCESS** | - Resource Availability - Could be a facilitator as well as a lack/barrier? i.e maternal resources, to access care, or resources at a facility or available to a visiting professional care giver - Capacity to access health facility or healthcare provider? It could be quite close, but hard to get to, or a long way away, but easy to get to. In some systems, women don’t go to health facility to access care, the healthcare provider comes to them - Place of postnatal care/delivery arrangements (including issues around integration of mother and baby care, and issues around the question of home visits) - Time/waiting times |
| **BEHAVIORS AND ATTITUDES** | - (Lack of) Respectful care - (Lack of) trust in the system - Belief (or not) in the need for postnatal care? - Fear of stigma/test results/ policy/child services taking child away/knowing something is wrong with herself or the baby - Value or otherwise of the mother/baby to the family/society (including not seeking care because babies are “weak”, not considered “important”, money not well spent if used for newborn care, etc.) |
| **EXTERNAL INFLUENCES** | - Influence of family/peers - Capacity for women to travel for care/Freedom of movement/quarantine - Influence of traditional/societal beliefs/superstitions (including social beliefs about postnatal care in modern and postmodern society that are not well grounded in evidence) |
| **WHAT WOMEN WANT AND NEED** | - Continuity of care/carer - Need for information/advice (recognition of danger signs) - Optimising health of the baby (including thriving and feeding) - Optimising health of the mother (physical, psychological, emotional support) - Support for effective transition to motherhood (confidence, competence and adapting to changes in self and relationships to others) |
